# Identification of a novel aromatic-turmerone analog that activates chaperone-mediated autophagy through the persistent activation of p38

**DOI:** 10.3389/fcell.2024.1418296

**Published:** 2024-08-08

**Authors:** Kensuke Motomura, Erika Ueda, Alex Boateng, Masaharu Sugiura, Keiichi Kadoyama, Natsuko Hitora-Imamura, Yuki Kurauchi, Hiroshi Katsuki, Takahiro Seki

**Affiliations:** ^1^ Department of Chemico-Pharmacological Sciences, Graduate School of Pharmaceutical Sciences, Kumamoto University, Kumamoto, Japan; ^2^ Graduate School of Pharmaceutical Sciences, Sojo University, Kumamoto, Japan; ^3^ Department of Pharmacology, Faculty of Pharmaceutical Sciences, Himeji-Dokkyo University, Himeji, Japan

**Keywords:** aromatic-turmerone, chaperone-mediated autophagy, Nrf2, p38, LAMP2A, HSC70

## Abstract

**Introduction:** Aromatic (Ar)-turmerone is a bioactive component of turmeric oil obtained from *Curcuma longa*. We recently identified a novel analog (A2) of ar-turmerone that protects dopaminergic neurons from toxic stimuli by activating nuclear factor erythroid 2-related factor 2 (Nrf2). D-cysteine increases Nrf2, leading to the activation of chaperone-mediated autophagy (CMA), a pathway in the autophagy-lysosome protein degradation system, in primary cultured cerebellar Purkinje cells. In this study, we attempted to identify novel analogs of ar-turmerone that activate Nrf2 more potently and investigated whether these analogs activate CMA.

**Methods:** Four novel analogs (A4–A7) from A2 were synthesized. We investigated the effects of A2 and novel 4 analogs on Nrf2 expression via immunoblotting and CMA activity via fluorescence observation.

**Results:** Although all analogs, including A2, increased Nrf2 expression, only A4 activated CMA in SH-SY5Y cells. Additionally, A4-mediated CMA activation was not reversed by Nrf2 inhibition, indicating that A4 activated CMA via mechanisms other than Nrf2 activation. We focused on p38, which participates in CMA activation. Inhibition of p38 significantly prevented A4-mediated activation of CMA. Although all novel analogs significantly increased the phosphorylation of p38 6 h after drug treatment, only A4 significantly increased phosphorylation 24 h after treatment. Finally, we revealed that A4 protected SH-SY5Y cells from the cytotoxicity of rotenone, and that this protection was reversed by inhibiting p38.

**Conclusion:** These findings suggest that the novel ar-turmerone analog, A4, activates CMA and protects SH-SY5Y cells through the persistent activation of p38.

## 1 Introduction

Aromatic (Ar)-turmerone is a bioactive component of turmeric oil obtained from *Curcuma longa* (Roth et al., 1998). It has been reported to exert anti-inflammatory activity in cultured microglia and glial cells in mice (Park et al., 2012c; 2012a; Chen et al., 2018). We recently demonstrated that ar-turmerone analogs inhibit dopaminergic neurodegeneration triggered by microglial activation and dopaminergic toxins in midbrain slice cultures without affecting microglial activation (Hori et al., 2021). These chemicals activate nuclear factor erythroid 2-related factor 2 (Nrf2), a transcription factor that regulates antioxidant proteins (He et al., 2020) in dopaminergic neurons. We proposed a novel mechanism (Nrf2 activation) for the neuroprotective effect of ar-turmerone and its analogs.

The autophagy-lysosome degradation system contributes to the maintenance of intracellular protein homeostasis (Ciechanover and Kwon, 2015; Aman et al., 2021). Chaperone-mediated autophagy (CMA) is one of three pathways involved in the delivery of protein substrates to lysosomes. In CMA, substrates containing the KFERQ motif are recognized by a molecular chaperone, heat shock cognate protein 70 kDa (Hsc70), and delivered into lysosomes via the oligomers of lysosome-associated membrane protein 2A (LAMP2A) (Kaushik and Cuervo, 2018). CMA is gaining attention as an etiological factor for various diseases, particularly Parkinson’s disease (PD) (Campbell et al., 2018). α-Synuclein frequently accumulates in the dopaminergic neurons in the substantia nigra. CMA is involved in the degradation of α-synuclein and is impaired by mutations that cause inherited PD (Cuervo et al., 2004). In addition, a reduction in Hsc70 and LAMP2A was observed in the *postmortem* brains and lymphocytes of patients with sporadic PD (Alvarez-Erviti et al., 2010; Wu et al., 2011; Sala et al., 2014). Furthermore, the shRNA-mediated knockdown of LAMP2A caused dopaminergic neurodegeneration in the substantia nigra of mice (Xilouri et al., 2016). Collectively, these findings suggest that CMA impairment contributes to the pathogenesis of PD.

Accumulating evidence has indicated that decreased CMA activity is commonly observed in cellular and animal models of neurodegenerative diseases, including Alzheimer’s disease, Huntington’s disease, and amyotrophic lateral sclerosis (Bourdenx et al., 2021; Kanno et al., 2022; Idera et al., 2023). We have previously demonstrated that CMA impairment is related to the pathogenesis of spinocerebellar ataxia (SCA), a group of autosomal dominant neurodegenerative diseases characterized by progressive cerebellar ataxia (Seki et al., 2012; Seki et al., 2018a; Sato et al., 2021). We recently identified D-cysteine as a candidate for common preventive drug for SCA. D-cysteine is converted to hydrogen sulfide in the cerebellum (Shibuya et al., 2013; Seki et al., 2018b) and prevents pathogenic phenotypes in primary cultured cerebellar Purkinje cells and SCA type 1 (SCA1) model mice (Ohta et al., 2021). It activates CMA in primary cultured Purkinje cells via the generation of hydrogen sulfide and activation of Nrf2 (Ueda et al., 2022). Therefore, the cytoprotective effect of Nrf2-activating chemicals may be mediated by CMA activation.

In the present study, we identified novel ar-turmerone analogs that activate Nrf2 more potently than the analogs that we have reported in the previous study (Hori et al., 2021) and investigated whether these novel analogs activate CMA in neuroblastoma SH-SY5Y cells. We identified a novel analog (A4) that activates both Nrf2 and CMA. However, the A4-triggered activation of CMA was not affected by the inhibition of Nrf2. We revealed that A4 induces persistent activation of p38, resulting in upregulation of LAMP2A and Hsc70 and activation of CMA.

## 2 Materials and methods

### 2.1 Materials

Mycophenolic acid (MPA, M5255), anti-β-actin mouse monoclonal antibody (A1978), and anti-β-tubulin mouse monoclonal antibody (T5201) were purchased from Sigma-Aldrich (St. Louis, MO, United States). The anti-human LAMP2A rabbit polyclonal antibody (ab125068), anti-TSG101 rabbit polyclonal antibody (ab125011), and anti-α-synuclein rabbit monoclonal antibody (ab138501) were obtained from AbCam (Cambridge, United Kingdom). Anti-Hsc70 mouse monoclonal IgM antibody (NB120–2788) was obtained from Novus Biologicals (Centennial, CO, United States). The anti-nuclear factor-erythroid 2-related factor 2 (Nrf2) rabbit polyclonal antibody (16396–1–AP) and anti-phospho-p38 (Thr180/Thy182) rabbit polyclonal antibody (28796–1–AP, for immunostaining) were purchased from ProteinTech (Rosemont, IL, United States). Anti-p38 rabbit polyclonal antibody (9212) and phospho-p38 (Thr180/Thy182) rabbit polyclonal antibody (9211, for immunoblotting) were obtained from Cell Signaling Technology (Danvers, MA, United States). Anti-Anti-heme oxygenese-1 (HO-1) rabbit polyclonal antibody (GTX101147) and anti-NAD(P)H quinone dehydrogenase 1 (NQO1) rabbit polyclonal antibody (GTX100235) were purchased from GeneTex, Irvine, CA, United States). HaloTag ligand fused with SaraFluor™ 650T Ligand (A308-01) was obtained from Goryo Chemical (Sapporo, Japan). Penicillin–streptomycin solution (26523–84) was obtained from Nacalai Tesque (Kyoto, Japan). pEBMulti-Neo (057–08131) and SB203580 (199–16551) were purchased from FujiFilm Wako Chemicals (Osaka, Japan). ML385 (S8798) was purchased from Selleck Chemicals (Houston, TX, United States). Cell counting kit-8 (CCK–8, 347–07621) was obtained from Dojindo Laboratories (Kumamoto, Japan). Oligo DNA primers were purchased from Integrated DNA Technologies (Coralville, IA, United States). The 8-well coverglass chamber (5232–008) was obtained from AGC Techno Grass (Tokyo, Japan). Lipofectamine 3000 (L3000008), BLOCK-iT™ Pol II miR RNAi Expression Vector Kit with EmGFP (K493600), and Alexa Fluor 488-conjugated donkey anti-rabbit IgG antibody (A–21206) were purchased from ThermoFisher Scientific (Waltham, MA, United States). The K4 transfection system was purchased from Biotex Laboratories (München, Germany). 4′, 6-Diamidino-2-phenylindole, dihydrochloride (DAPI, #4083) was purchased from Cell Signaling Technology (Danvers, MA, United States).

### 2.2 Synthesis of ar-turmerone analogs

**FIGURE 1:**
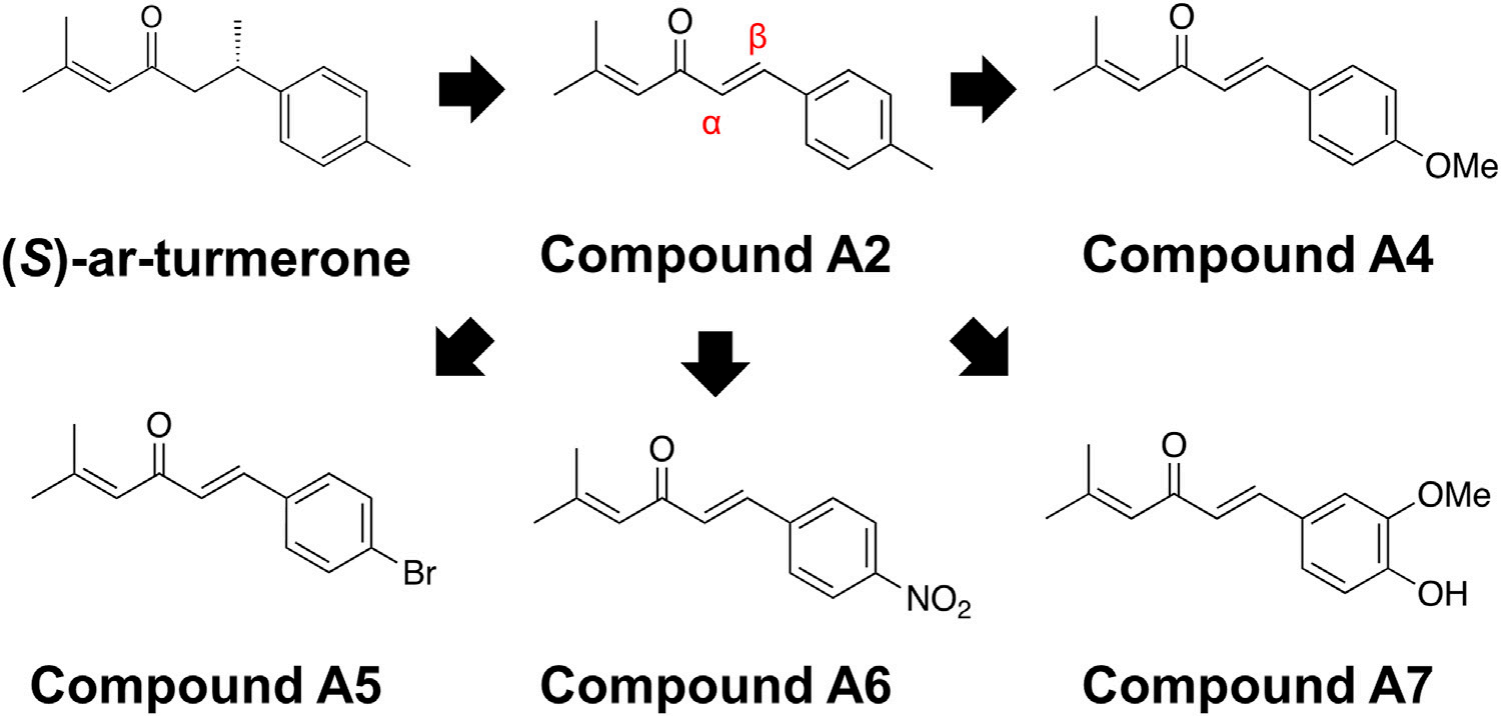
Chemical structures of ar-turmerone analogs. Compound A2 was generated from (S)-ar-turmerone as previously described. α and β carbons of A2 are indicated by the red characters. We synthesized A4–A7 to attach electron-donating and -withdrawing groups to the aromatic ring of A2.

Figure 1 illustrates the chemical structures of ar-turmerone and its analogs used in the present study.

#### 2.2.1 (*E*)-5-Methyl-1-(*p*-tolyl)hexa-1,4-dien-3-one (A2)

Compound A2 was prepared using a previously reported method (Kohler and Chadwell, 1922). A solution of NaOH (120 mg, 3 mmol) in H_2_O (1.2 mL) was added to a solution of mesityl oxide (229 μL, 2.0 mmol) in EtOH (0.8 mL). After stirring at room temperature (rt) for 5 min, p-tolualdehyde (236 μL, 2.0 mmol) was added to the mixture. The mixture was stirred at rt for 24 h and then extracted with Et_2_O (3 × 20 mL). The combined organic layers were dried over anhydrous Na_2_SO_4_, filtered, and concentrated under a vacuum. The residue was purified by column chromatography on silica gel (hexane/CH_2_Cl_2_ = 1:1) to yield A2 (210 mg, 52% yield).

TLC: R_f_ 0.22 (hexane/CH_2_Cl_2_ = 1:1, stained blue-green with phosphomolybdic acid). ^1^H NMR (500 MHz, CDCl_3_) δ 7.55 (d, *J* = 16.0 Hz, 1H), 7.45 (d, *J* = 8.0 Hz, 2H), 7.19 (d, *J* = 8.0 Hz, 2H), 6.75 (d, *J* = 16.0 Hz, 1H), 6.35–6.33 (m, 1H), 2.37 (s, 3H), 2.22 (s, 3H), 1.97 (s, 3H).

#### 2.2.2 (*E*)-1-(4-Methoxyphenyl)-5-methylhexa-1,4-dien-3-one (A4)

Compound A4 was prepared using a previously reported method (Kohler and Chadwell, 1922). A solution of NaOH (120 mg, 3 mmol) in H_2_O (1.2 mL) was added to a solution of mesityl oxide (229 μL, 2.0 mmol) in EtOH (0.8 mL). After stirring at rt for 5 min, p-anisaldehyde (243 μL, 2.0 mmol) was added to the mixture. The mixture was stirred at rt for 2 h and then extracted with Et_2_O (3 × 20 mL). The combined organic layers were dried over anhydrous Na_2_SO_4_, filtered, and concentrated under a vacuum. The residue was purified by column chromatography on silica gel (toluene/hexane = 20:1) to obtain A4 (95 mg, 22% yield).

TLC: R_f_ 0.16 (toluene/hexane = 20:1, stained black with phosphomolybdic acid). ^1^H NMR (Harmata et al., 2004) (500 MHz, CDCl_3_) δ 7.53 (d, *J* = 16.0 Hz, 1H), 7.51 (d, *J* = 8.6 Hz, 2H), 6.91 (d, *J* = 8.6 Hz, 2H), 6.68 (d, *J* = 16.0 Hz, 1H), 6.34–6.32 (m, 1H), 3.84 (s, 3H), 2.21 (d, *J* = 1.2 Hz, 3H), 1.96 (d, *J* = 1.2 Hz, 3H).

#### 2.2.3 (*E*)-1-(4-Bromophenyl)-5-methylhexa-1,4-dien-3-one (A5)

Compound A5 was prepared using a previously reported method (Kohler and Chadwell, 1922). A solution of NaOH (120 mg, 3 mmol) in H_2_O (1.2 mL) was added to a solution of mesityl oxide (345 μL, 3.0 mmol) in EtOH (1.8 mL). After stirring at rt for 5 min, p-bromobenzaldehyde (370 mg, 2.0 mmol) was added to the mixture. The mixture was stirred at rt for 3 h and then extracted with Et_2_O (3 × 20 mL). The combined organic layers were dried over anhydrous Na_2_SO_4_, filtered, and concentrated under a vacuum. The residue was purified by column chromatography on silica gel (toluene/hexane = 2:1) to obtain A5 (127 mg, 24% yield).

TLC: R_f_ 0.32 (toluene/hexane = 2:1, stained black with phosphomolybdic acid). ^1^H NMR (500 MHz, CDCl_3_) δ 7.51 (d, *J* = 8.6 Hz, 2H), 7.49 (d, *J* = 16.0 Hz, 1H), 7.41 (d, *J* = 8.6 Hz, 2H), 6.76 (d, *J* = 16.0 Hz, 1H), 6.33–6.32 (m, 1H), 2.22 (d, *J* = 1.2 Hz, 3H), 1.97 (d, *J* = 1.2 Hz, 3H).

#### 2.2.4 (*E*)-5-Methyl-1-(4-nitrophenyl)hexa-1,4-dien-3-one (A6)

Compound A6 was prepared using a previously reported method (Sugiura et al., 2015). TiCl_4_ (2.94 M in CH_2_Cl_2_; 0.88 mL, 2.6 mmol) and Bu_3_N (0.67 mL, 2.8 mmol) were successively added to a stirred solution of mesityl oxide (252 μL, 2.2 mmol) in CH_2_Cl_2_ (6.0 mL) at −78°C. After 30 min, p-nitrobenzaldehyde (302 mg, 2.0 mmol) was added in one portion to the mixture at −78°C. Formation of the aldol product was detected by TLC analysis. After 2 h, pyridine (0.81 mL, 10 mmol) was added at −78°C and the reaction mixture was warmed to rt by removing it from the cooling bath. After stirring at rt for 4 h, the reaction mixture was diluted in Et_2_O (5 mL). The mixture was filtered through a Celite pad and the filtrate was concentrated. The residue was crystallized by adding methanol/water (5:1). The crystalline product was then collected by filtration and dried under vacuum to obtain A6 (260 mg, 56% yield). The filtrate was evaporated and purified by column chromatography on silica gel (hexane/CH_2_Cl_2_ = 1:2) to obtain A6 (143 mg, 31% yield).

TLC: R_f_ 0.33 (hexane/CH_2_Cl_2_ = 1:2, stained black with phosphomolybdic acid). ^1^H NMR (500 MHz, CDCl_3_) δ 8.25 (d, *J* = 8.6 Hz, 2H), 7.69 (d, *J* = 8.6 Hz, 2H), 7.58 (d, *J* = 16.0 Hz, 1H), 6.88 (d, *J* = 16.0 Hz, 1H), 6.36–6.34 (m, 1H), 2.25 (d, *J* = 1.2 Hz, 3H), 2.00 (d, *J* = 1.2 Hz, 3H).

#### 2.2.5 (*E*)-1-(4-Hydroxy-3-methoxyphenyl)-5-methylhexa-1,4-dien-3-one (A7)

Compound A7 was prepared using a previous reported method (Narender and Papi Reddy, 2007) BF_3_·OEt_2_ (0.13 mL, 1.0 mmol) was gradually added to a mixture of mesityl oxide (230 μL, 2.0 mmol) and vanillin (311 mg, 2.0 mmol) at rt. The mixture was then stirred for 2 h at rt. After dilution with EtOAc (40 mL), the mixture was washed with water (3 × 10 mL). The organic layer was dried over anhydrous Na_2_SO_4_, filtered, and concentrated under a vacuum. The residue was purified by column chromatography on silica gel (hexane/CH_2_Cl_2_ = 1:10) to obtain A7 (88 mg, 19% yield).

TLC: R_f_ 0.33 (hexane/CH_2_Cl_2_ = 1:10, stained black with phosphomolybdic acid). ^1^H NMR (500 MHz, CDCl_3_) δ 7.50 (d, *J* = 16.0 Hz, 1H), 7.10 (dd, *J* = 8.0, 1.7 Hz, 1H), 7.06 (d, *J* = 1.7 Hz, 1H), 6.92 (d, *J* = 8.6 Hz, 1H), 6.65 (d, *J* = 16.0 Hz, 1H), 6.35–6.33 (m, 1H), 5.94 (s, 1H), 3.93 (s, 3H), 2.21 (d, *J* = 1.2 Hz, 3H), 1.96 (d, *J* = 1.2 Hz, 3H).

### 2.3 Cell culture

SH-SY5Y cells were purchased from Riken Cell Bank (Tsukuba, Japan) and cultured in Dulbecco’s modified Eagle medium/F-12 mixture (1:1) medium, supplemented with 10% fetal bovine serum (FBS), 100 units/mL penicillin, and 100 μg/mL streptomycin in a humidified atmosphere containing 5% CO_2_ at 37°C (Seki et al., 2010). Glyceraldehyde-3-phosphate dehydrogenase fused with HaloTag (GAPDH-HT) cDNA was constructed as previously described (Seki et al., 2012) and subcloned into pEBMulti-Neo (FujiFilm Wako Chemicals, Osaka, Japan), an episomal vector for establishing stable cell lines (Shibata et al., 2007). Cells were transfected with GAPDH-HT in pEBMulti-Neo using Lipofectamine 3000 and cultured in the presence of G418 (0.5 mg/mL) to select the transfected cells.

### 2.4 Assessment of CMA and microautophagy (mA) activities

We investigate the effects of the chemicals on CMA/mA activity using our original activity marker for CMA/mA, GAPDH-HT (Seki et al., 2012; Sato et al., 2016). SH-SY5Y cells stably expressing GAPDH-HT were placed onto an 8-well coverglass chamber (1 × 10^4^ cells/well). The next day, the cells were incubated with the HT ligand fused with SaraFluor 650T (0.5 μM) for 10 min at 37°C to label GAPDH-HT with a fluorescent dye (SaraFluor 650T). After washing with culture media, the cells were cultured with the culture media containing 0.2% DMSO (vehicle), 5 μM MPA, or 20 μM ar-turmerone analogs (A2, A4–A7) for 24 h. In several experiments, cells were treated with A4, simultaneously with 1 μM ML385 (an Nrf2 inhibitor) or 10 μM SB203580 (a p38 inhibitor). After fixing with 4% paraformaldehyde, fluorescence of SaraFluor 650T in cells was observed using the THUNDER imaging system (Leica Microsystems, Wetzlar, Germany). The background noise of the fluorescent images was subtracted using Instant Computational Cleaning (Leica Microsystems). The CMA/mA activity was assessed by counting the number of GAPDH-HT puncta in the cytoplasm.

To investigate the effects of these chemicals on CMA and mA separately, we conducted miRNA-mediated knockdown of LAMP2A or TSG101 and evaluated the GAPDH-HT puncta in transfected cells. Plasmids to express GFP and miRNA (negative control, LAMP2A, or TSG101) were constructed using a BLOCK-iT™ Pol II miR RNAi Expression Vector Kit with EmGFP as previously described (Sato et al., 2021). Target sequences of miRNA were as follows; negative control miRNA: 5′-GTC​TCC​ACG​CGC​AGT​ACA​TTT-3′, LAMP2A miRNA: 5′-TAT​TCT​AGT​GTT​GCT​GGC​TTA-3, TSG101 miRNA: 5′- GCG​GAT​GAA​GGA​GGA​AAT​GGA-3′. GFP-positive cells were regarded as cells expressing miRNAs. SH-SY5Y cells were transfected with these plasmids using the K4 transfection system according to the manufacturer’s protocol. Two days after transfection, cells on the 8-well coverglass chamber were labeled with SaraFluor 650T-HT ligand, followed by the treatment with vehicle or 20 μM A4 for 24 h. Fluorescence images of GFP and SaraFluor-labeled GAPDH-HT of GFP-positive cells were obtained using the THUNDER imaging system. CMA activity was represented by GAPDH-HT puncta in cells expressing TSG101 miRNA, whereas mA activity was represented by puncta in cells expressing LAMP2A miRNA.

### 2.5 Immunoblotting

Immunoblotting was conducted as previously described (Idera et al., 2023). Briefly, SH-SY5Y cells stably expressing GAPDH-HT were placed onto 12-well plates (2 × 10^5^ cells/well). The following day, cells were treated with vehicle, 5 μM MPA, or 20 μM ar-turmerone analogs for 6 or 24 h and were lysed in radioimmunoprecipitation assay (RIPA) buffer, as previously described. The protein concentrations in the cell lysates were quantified using a bicinchoninic acid assay. Equal amounts of proteins from the cell and cerebellar lysates were subjected to SDS-PAGE, followed by immunoblot analyses using anti-Nrf2 (1:1,000), anti-HO-1 (1:1,000), NQO-1 (1:2000), anti-p38 (1:1,000), anti-phospho-p38 (Thr180/Tyr182), anti-LAMP2A (1:2,000), anti-Hsc70 (1:2,000), anti-α-synuclein (1:1,000), anti-β-actin (1:5,000), and β-tubulin (1:5,000) antibodies as primary antibodies. Immunoreactive bands were quantified using ImageJ software. Protein amounts were normalized by relative amounts to β-actin or β-tubulin.

### 2.6 Immunostaining

Immunostaining was conducted as previously described (Ogawa et al., 2013). Briefly, SH-SY5Y cells on the 8-well coverglass chamber were treated with vehicle or 20 μM A4 for 24 h. After fixation, cells were immunostained with an anti-phospho-p38 (Thr180/Tyr182) antibody (1:500), followed by incubation with a buffer containing Alexa Fluor 488-conjugated anti-rabbit IgG antibody (1:500) and DAPI (500 ng/mL) for nuclear staining. Fluorescence images of Alexa Fluor 488 and DAPI were obtained using the THUNDER imaging system. The immunofluorescence intensities of phosphorylated p38 in whole cells and nuclei were quantified using the ImageJ software (National Institutes of Health, Bethesda, MD, United States).

### 2.7 Reverse transcription-quantitative polymerase chain reaction (RT-qPCR)

SH-SY5Y cells stably expressing GAPDH-HT were placed onto 12-well plates and treated with the chemicals for 24 h, as described in Section 2.5. Total RNA was extracted from each well using NucleoSpin RNA Plus (catalog no. 740984.50; Machery-Nagel, Düren, Germany). RT was performed using the ReverTra Ace qPCR-RT kit (catalog no. FSQ-101; TOYOBO, Tokyo, Japan) containing equal amounts of total RNA. Real-time qPCR was performed using THUNDERBIRD Next SYBR qPCR Mix (catalog no. QPX-201, TOYOBO) on a 7500 Fast Real-Time PCR System (Applied Biosystems, Waltham, MA, United States). The primer sequences are listed in Table 1. The reactions were quantified by selecting the amplification cycle when the PCR product of interest was first detected (the threshold cycle). Data were analyzed using the comparative threshold cycle method. The β-actin mRNA level was used as the internal control for each sample.

**TABLE 1:** Primer sequences used in RT-qPCR analyses.

Gene name	Primer sequences (5′→3′)	Reference
LAMP2A	Forward: GGGTTCAGCCTTTCAATGTG Reverse: CAGCATGATGGTGCTTGAGA	Nishikawa et al. (2020)
LAMP2B	Forward: GGGTTCAGCCTTTCAATGTG Reverse: CCTGAAAGACCAGCACCAAC	Nishikawa et al. (2020)
LAMP1	Forward: CTGCCTTTAAAGCTGCCAAC Reverse: TGTTCTCGTCCAGCAGACAC	Ivanova et al. (2019)
HSPA8 (Hsc70)	Forward: GACAACCGAATGGTCAAC Reverse: GTACGGAGGCGTCTTACA	Qin et al. (2023)
TFEB	Forward: GCGGCAGAAGAAAGACAATC Reverse: CTGCATCCTCCGGATGTAAT	Gardiner et al. (2013)
MEF2D	Forward: AACGCCGACATCATCGAG Reverse: GGCTCTGTTCCAGCGAGT	Rusmini et al. (2019)
ACTB (β-actin)	Forward: CATCGTCCACCGCAAATGCTTC Reverse: AACCGACTGCTGTCACCTTCAC	Wang et al. (2021)

### 2.8 Cell survival assay

SH-SY5Y cells stably expressing GAPDH-HT were placed onto 96-well plates (4 × 10^3^ cells/well). The following day, the cells were treated with several chemicals in the presence or absence of 10 μM rotenone, a mitochondrial toxin that induces cell death. After 24 h of cultivation, the number of surviving cells in each well was determined using Cell Counting Kit-8 (catalog No. 347–07621, Dojo Laboratories, Kumamoto, Japan), according to the manufacturer’s protocol (Tominaga et al., 1999).

Rotenone-triggered cytotoxicity was also evaluated by the nuclear condensation, a morphological hallmark of apoptosis. SH-SY5Y cells transfected with plasmids to express GFP and miRNA (negative control or LAMP2A) were spread onto 8-well coverglass chamber (2 × 10^4^ cells/well), followed by the treatment with vehicle (0.2% DMSO) or 20 μM A4 in the presence or absence of 10 μM rotenone for 24 h. After the After fixing with 4% paraformaldehyde, cells were stained with 500 ng/mL DAPI, followed by the fluorescence observation of GFP and DAPI using the THUNDER imaging system. Cytotoxicity of GFP-positive cells were evaluated by the percentage of cells with condensed nuclei in 50–200 GFP positive cells per well.

### 2.9 Statistical analyses

Quantitative data were obtained from more than three independent experiments. Most quantitative data are presented as percentages of vehicle-treated cells. Statistical differences in quantitative data from the immunoblot experiments and the apoptotic assay (nuclear condensation) were determined using one-way ANOVA, followed by Tukey’s multiple comparison tests. Statistical differences in quantitative data for GAPDH-HT and cell survival analyses were determined using Kruskal–Wallis tests, followed by Dunn’s multiple comparison tests. Statistical differences in the quantitative data from immunostaining analyses were determined using an unpaired *t*-test. Statistical analyses were performed using the GraphPad Prism six software (GraphPad Software, San Diego, CA, United States). Significance was set at *p* < 0.05.

## 3 Results

### 3.1 Effects of ar-turmerone analogs on Nrf2 in SH-SY5Y cells

**FIGURE 2:**
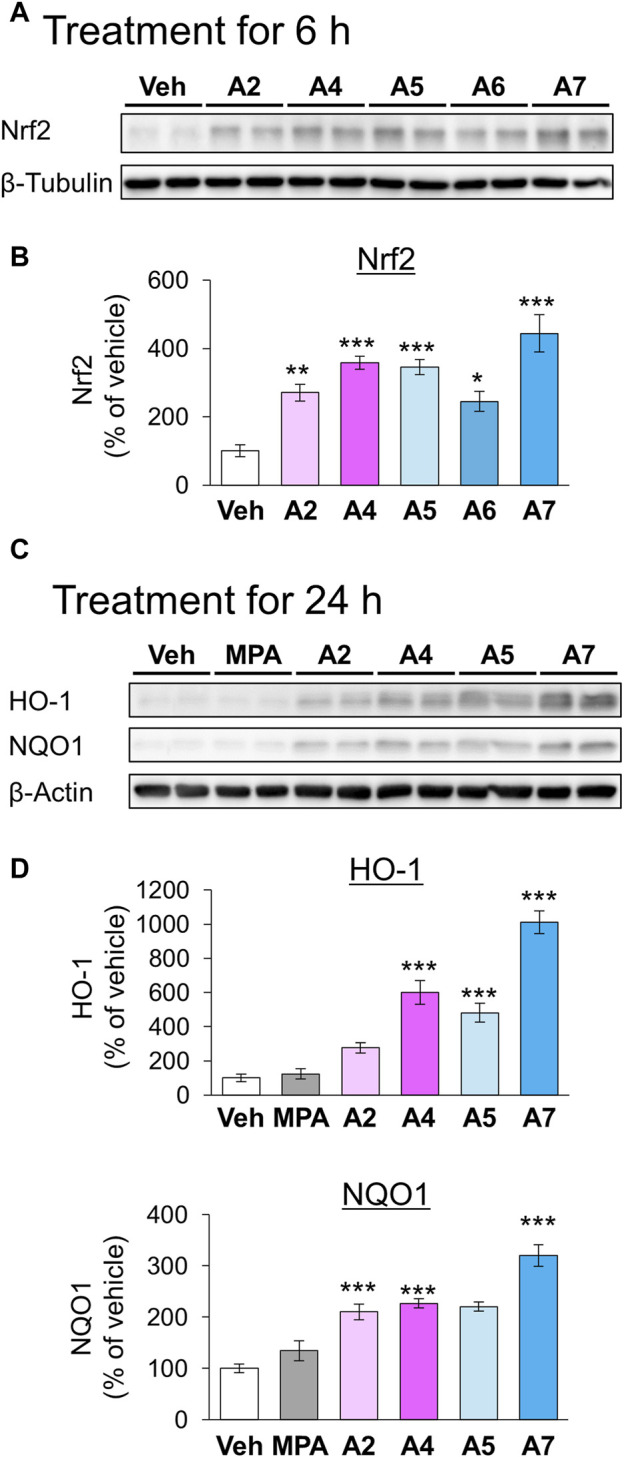
Effects of ar-turmerone analogs on the expression of Nrf2 and its target proteins in SH-SY5Y cells stably expressing GAPDH-HT. **(A)** Representative immunoblot images of Nrf2 and β-tubulin in cells treated with vehicle (0.1% DMSO) or ar-turmerone analogs (20 μM) for 6 h. **(B)** Quantitative analyses of immunoreactive bands of Nrf2. The band intensities were normalized with the bands of β-tubulin as an internal control. Data are presented as the mean ± SEM of four different samples. **(C)** Representative immunoblot images of HO-1, NQO1, and β-actin in cells treated with vehicle, mycophenolic acid (MPA, 5 μM), or ar-turmerone analogs (20 μM) for 24 h. **(D)** Quantitative analyses of immunoreactive bands of HO-1 and NQO1. The band intensities were normalized with the bands of β-actin as an internal control. Data are presented as the mean ± SEM of eight different samples. *p < 0.05, **p < 0.01, ***p < 0.001 vs. vehicle (one-way ANOVA, followed by a *post hoc* Tukey test).

We have previously demonstrated that the electrophilicity of β carbon in ar-turmerone analogs is important for the activation of Nrf2 (Figure 1). Therefore, we synthesized four novel analogs (A4–A7) from A2, the most potent ar-turmerone analog reported previously (Hori et al., 2021). To increase the electrophilicity of β carbon, we added electron-donating and -withdrawing groups to the aromatic ring of A2 (Figure 1). We investigated the effects of A2 and novel ar-turmerone analogs on Nrf2 expression in SH-SY5Y cells. After 6 h of treatment at a concentration of 20 μM, all 5 compounds significantly increased Nrf2 in SH-SY5Y cells (Figures 2A,B). Among them, A7 increased Nrf2 most potently, and A4 and A5 induced higher Nrf2 expression than A2. We also evaluated the downstream targets of Nrf2 (HO-1 and NQO1) in response to 24-h treatment with ar-turmerone analogs. We excluded A6 because of its high toxicity in SH-SY5Y cells after 24 h of treatment. MPA was used as a positive control for CMA activation. Compounds A4, A5, and A7 significantly increased the expression of HO-1 and NQO1 (Figures 2C,D). The abilities of compounds to induce the expression of Nrf2 target proteins correlated with the Nrf2 levels elevated by these compounds. Taken together, these findings indicated that the three ar-turmerone analogs activated CMA more potently than A2, and the most potent analog was A7.

### 3.2 Effects of ar-turmerone analogs on CMA and mA activity

**FIGURE 3:**
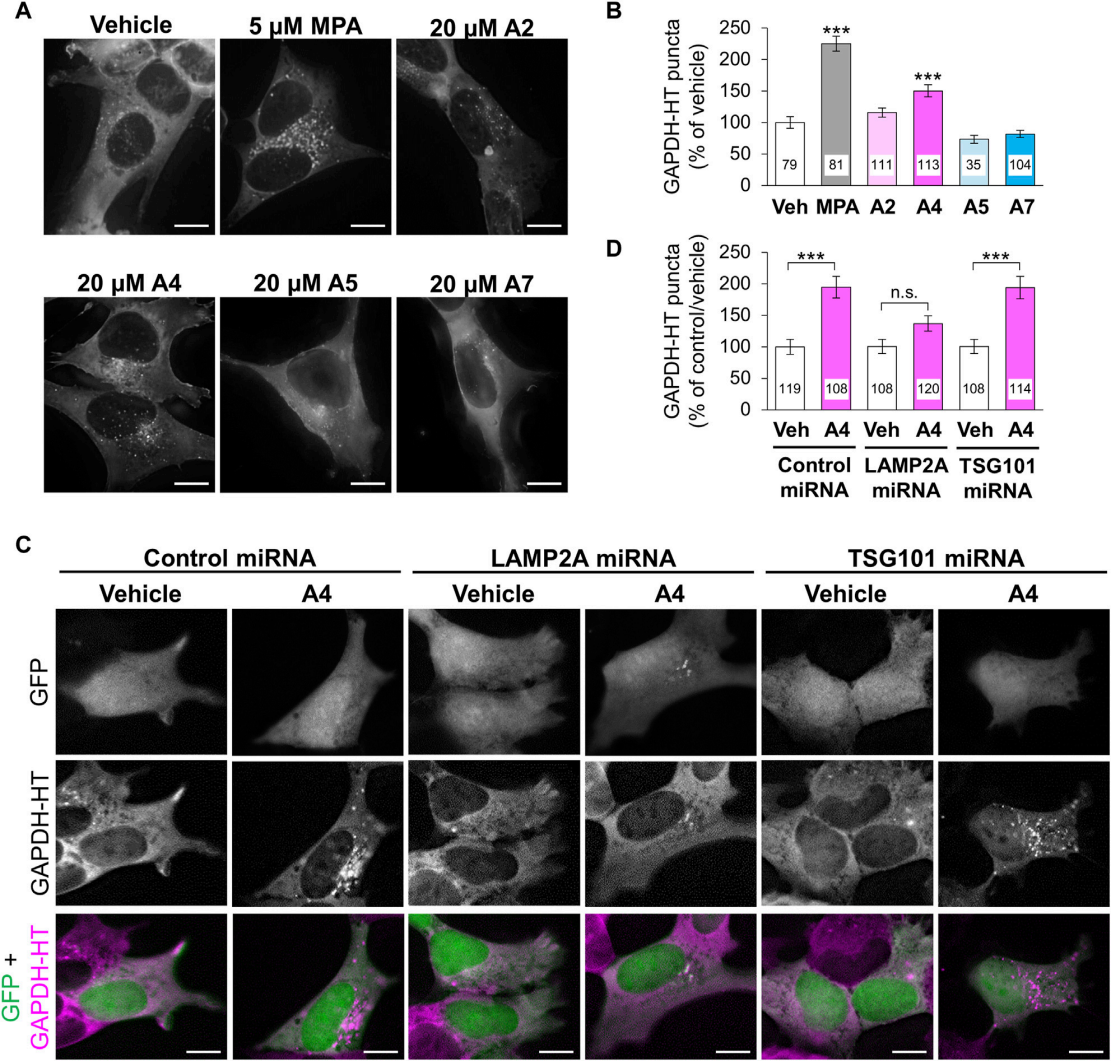
Effects of ar-turmerone analogs on CMA/mA activity in SH-SY5Y cells stably expressing GAPDH-HT. **(A)** Representative fluorescence image of SaraFluor 650T attached to GAPDH-HT in cells treated with vehicle, MPA (5 μM), or ar-turmerone analogs (20 μM) for 24 h. **(C)** Representative fluorescence image of GFP and SaraFluor 650T attached to GAPDH-HT and merged images of GFP (green) and SaraFluor (magenta) in cells treated with vehicle or A4 (20 μM) for 24 h. Cells expressing GFP means cells expressing miRNA (negative control miRNA, LAMP2A miRNA, or TSG101 miRNA). Scale bars = 10 μm. **(B, D)** Quantitative analyses of GAPDH-HT puncta per cell shown in A and C, respectively. Data are represented as the percentage of GAPDH-HT puncta in cells treated with vehicle **(B)** and in cells transfected with control miRNA and treated with vehicle **(D)**. Numbers in the columns represent the number of observed cells. n.s., not significant, ***p < 0.001 (Kruskal–Wallis test, followed by a *post hoc* Dunn’s test).

Next, we investigated the effects of these ar-turmerone analogs on CMA and mA activity. In mA that is another pathway involved in autophagy-lysosomal protein degradation, Hsc70 is also involved in the recognition of protein substrates (Seki and Katsuki, 2021). Hsc70-substrate complexes invaginate late endosomes and are delivered to lysosomes through the fusion of late endosomes with lysosomes (Sahu et al., 2011). We previously established a method to assess CMA and mA activity using a CMA/mA activity marker, GAPDH fused with HaloTag (GAPDH-HT), which is recognized by Hsc70 and translocated from the cytoplasm to late endosomes and lysosomes via CMA and mA (Seki et al., 2012; Sato et al., 2016). We constructed SH-SY5Y cells stably expressing GAPDH-HT and monitored punctate accumulation, which represents CMA and mA activity. After 24 h of treatment, MPA and A4 significantly increased punctate accumulation of GAPDH-HT (Figures 3A,B). A2 tended to increase its accumulation (approx. 15%). Unexpectedly, A7 did not increase punctate accumulation despite having the highest potency to activate Nrf2.

To determine whether CMA or mA were activated by A4, we evaluated punctate accumulation of GAPDH-HT following the miRNA-mediated knockdown of LAMP2A, a CMA-related protein, and TSG101, an mA-related protein (Sato et al., 2016). We used plasmids (pcDNA™6.2-GW/EmGFP-miR) that express GFP and miRNA in the same cells to visualize the miRNA-expressing cells. We transiently transfected plasmids expressing GFP and miRNA (negative control, LAMP2A, or TSG101) to SH-SY5Y cells stably expressing GAPDH-HT. Because GFP was expressed in approximately 10% of cells, it was difficult to evaluate the knockdown efficiency of miRNAs by immunoblot analysis in SH-SY5Y cells. Therefore, we conducted immunofluoresncece analyses to evaluate the knockdown of LAMP2A and TSG101. We observed that LAMP2A immunoreactivity was prominently decreased in GFP and LAMP2A miRNA-expressing cells, compared with surrounding unexpressed cells (Supplementary Figure S1), while it was not affected in cells expressing control miRNA and TSG101 miRNA-expressing cells. However, we unfortunately found good antibodies against TSG101 for the evaluation of TSG101 expression in the immunofluorescence analyses in SH-SY5Y cells. To determine the knockdown efficiency by immunoblotting, we transient transfected these plasmids into AD293 cells derived from human embryonic kidney cells. GFP was expressed in approximately 30% of AD293 cells (Supplementary Figures S2A, B). Immunoblot analyses of cell lysates from AD293 cells revealed that the amounts LAMP2A and TSG101 showed 30% decreases in cells transfected with plasmids expressing LAMP2A and TSG101, respectively, compared with those in cells transfected with control miRNA (Supplementary Figures S2C, D). The reduced ratio of the protein expression was equivalent to the ratio of miRNA-expressing cells. These results indicate that these miRNAs effectively reduced protein expression in the human-derived cell lines. A4 significantly increased the punctate accumulation of GAPDH-HT in SH-SY5Y cells expressing control or TSG101 miRNA. However, A4 failed to increase its accumulation in cells expressing LAMP2A miRNA (Figures 3C,D), indicating that A4 mainly activates CMA in SH-SY5Y cells.

### 3.3 Molecular mechanism of CMA activation by A4

**FIGURE 4:**
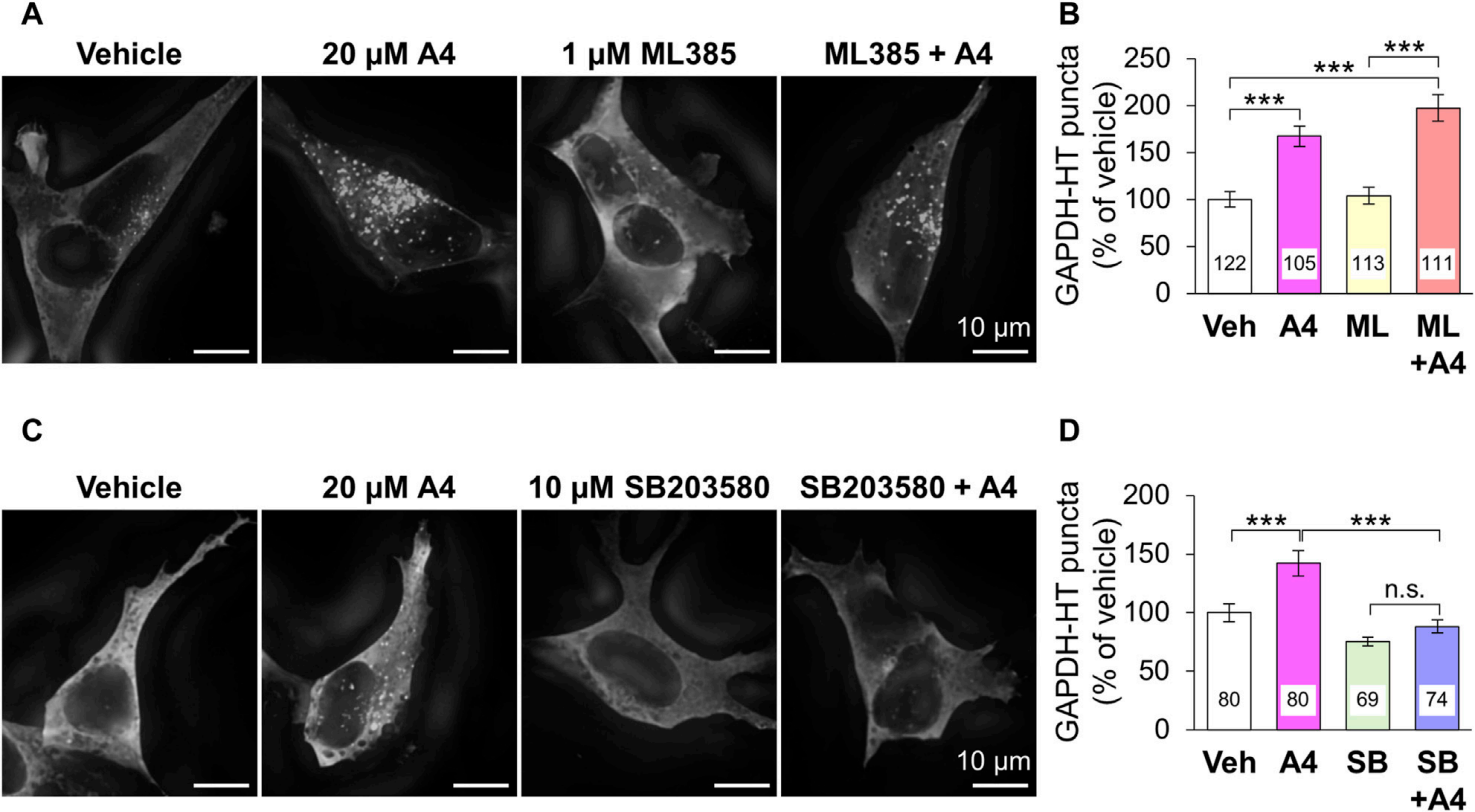
Effect of the inhibition of Nrf2 and p38 on A4-induced CMA activation in SH-SY5Y cells stably expressing GAPDH-HT. **(A, C)** Representative fluorescence image of SaraFluor 650T attached to GAPDH-HT in cells treated with vehicle or A4 (20 μM) in the absence or presence of 1 μM ML385 **(A)** and 10 μM SB203580 **(C)** for 24 h. Scale bars = 10 μm. **(B, D)** Quantitative analyses of GAPDH-HT puncta per cell shown in A and C, respectively. Data are represented as the percentage of GAPDH-HT puncta in cells treated with vehicle. Numbers in the columns represent the numbers of observed cells. n.s., not significant, ***p < 0.001 (Kruskal–Wallis test, followed by a *post hoc* Dunn’s test).

The ability of ar-turmerone analogs to activate Nrf2 did not correlate with their ability to activate CMA. In addition, simultaneous treatment with A4 and ML385, an Nrf2 inhibitor, did not affect the CMA activation triggered by A4 (Figures 4A,B). These findings suggested that CMA activation by A4 is not mediated by Nrf2.

**FIGURE 5:**
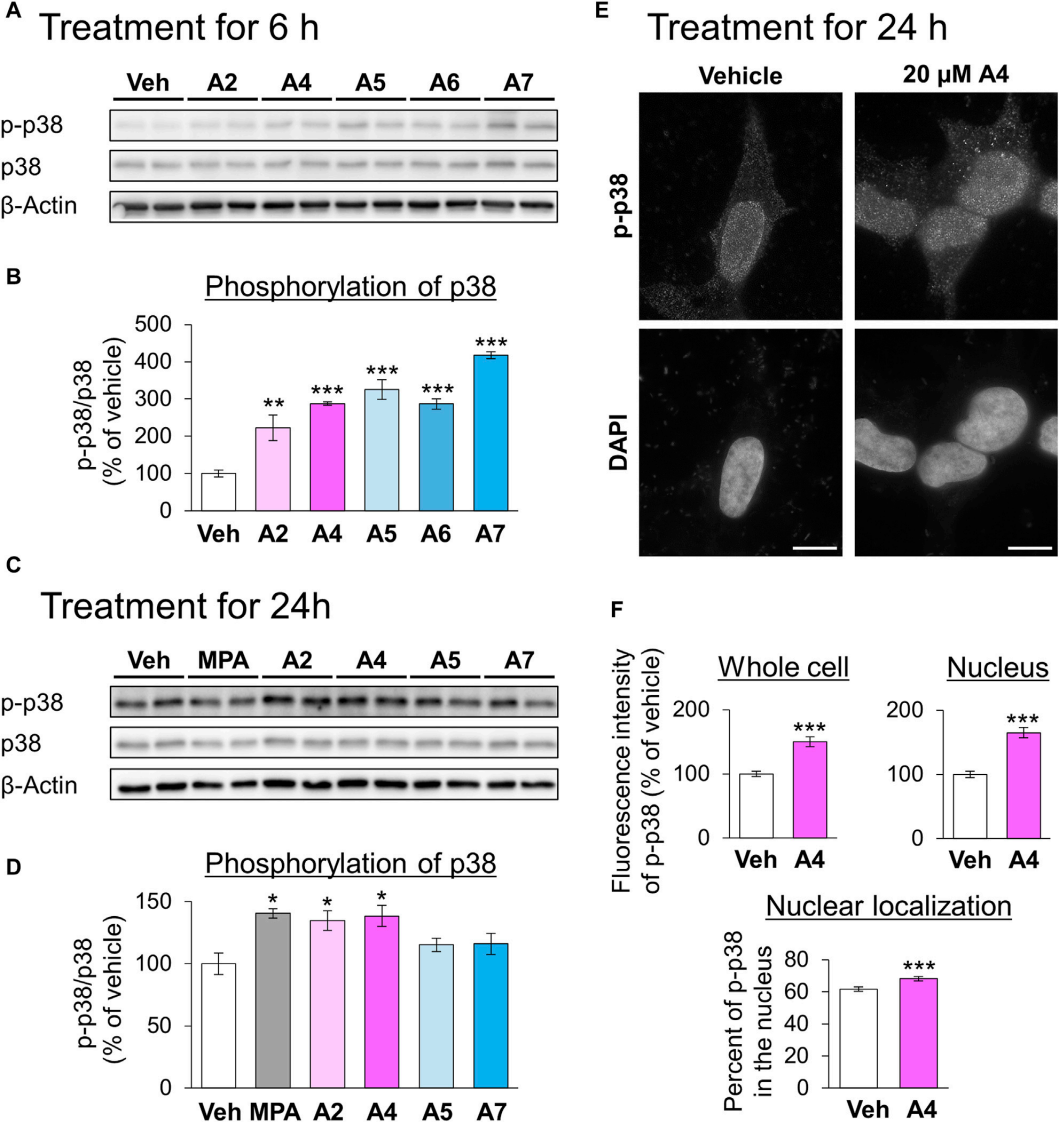
Effects of ar-turmerone analogs on the phosphorylation of p38 in SH-SY5Y cells stably expressing GAPDH-HT. **(A, C)** Representative immunoblot images of phosphorylated p38 (p-p38), p38, and β-actin in cells treated with vehicle, 5 μM MPA, or ar-turmerone analogs (20 μM) for 6 h **(A)** and for 24 h **(C)**. **(B, D)** Quantitative analyses of the phosphorylated ratio of p38 (p-p38/p38) after the 6-h treatment **(B)** and 24-h treatment **(D)**. Data are presented as the mean ± SEM of four different samples. *p < 0.05, **p < 0.01, ***p < 0.001 vs. vehicle (one-way ANOVA, followed by a *post hoc* Tukey test). **(E)** Representative fluorescence images of p-p38 immunoreactivity and DAPI in cells treated with vehicle or 20 μM A4 for 24 h. Scale bars = 10 μm. **(F)** Quantitative analyses of the immunofluorescence intensity of phosphorylated p38 in the whole cell and in the nucleus. From these values, we calculated the percentage of phosphorylated p38 in the nucleus. Data are presented as the mean ± SEM of 35 different cells. ***p < 0.001 vs. vehicle (unpaired *t*-test).

Next, we focused on the relationship between ar-turmerone analogs and p38, which is involved in the CMA activation by the endoplasmic reticulum (ER) stress (Li et al., 2017). The A4-induced activation of CMA was significantly reversed by simultaneous treatment with SB203580, a p38 inhibitor (Figures 4C,D). We investigated whether ar-turmerone analogs activate p38 by assessing their phosphorylation at Thr180 and Tyr182, which are localized in the activation loop of p38 and are phosphorylated in response to various stimulations to activate p38 (Kumar et al., 2018). After 6 h of treatment, all ar-turmerone analogs significantly enhanced the phosphorylation of p38 (Figures 5A,B). The most potent effect was induced by A7, similar to the Nrf2 activation. In contrast, MPA, A2, and A4 significantly increased p38 phosphorylation 24 h after treatment, but A5 and A7 did not (Figures 5C,D). These findings indicate that A2 and A4 persistently activate p38, whereas A5 and A7 strongly but transiently activate p38 in SH-SY5Y cells. In addition to the significant activation of CMA by A4, A2 tended to increase the punctate accumulation of GAPDH-HT (Figure 3). Therefore, persistent activation of p38 contributed to the activation of CMA by the ar-turmerone analogs A2 and A4.

We explored the changes in the localization of phosphorylated p38 in response to treatment with A4 for 24 h using immunostaining. Phosphorylated p38 was mainly localized to the nucleus of vehicle-treated cells (Figure 5E). A4 treatment significantly increased the amount of phosphorylated p38 in the whole cell and nucleus, and the nuclear localization of phosphorylated p38 (Figures 5E,F). These findings suggested that the increase in phosphorylated p38 in the nucleus was related to CMA activation.

### 3.4 Effects of ar-turmerone analogs on CMA-related proteins

**FIGURE 6:**
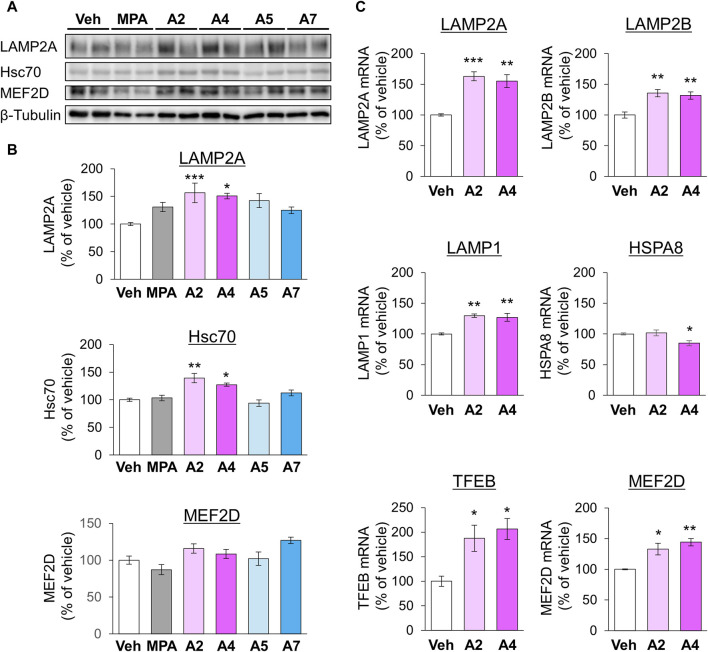
Effects of ar-turmerone analogs on the expression of CMA-related proteins and mRNAs in SH-SY5Y cells stably expressing GAPDH-HT. **(A)** Representative immunoblot images of LAMP2A, Hsc70, MEF2D, and β-tubulin in cells treated with vehicle, 5 μM MPA, or ar-turmerone analogs (20 μM) for 24 h. **(B)** Quantitative analyses of the immunoreactive bands of LAMP2A, Hsc70, and MEF2D. The band intensities were normalized with the bands of β-tubulin as an internal control. Data are presented as the mean ± SEM of four different samples. *p < 0.05, **p < 0.01, ***p < 0.001 vs. vehicle (one-way ANOVA, followed by a *post hoc* Tukey test). **(C)** The mRNA amounts of LAMP2A, LAMP2B, LAMP1, HSPA8 (Hsc70), TFEB, and MEF2D were quantified with RT-qPCR. Cells were treated with vehicle, 20 μM A2, or 20 μM A4 for 24 h. The data are presented as the mean ± SEM of four independent samples. *p < 0.05, **p < 0.01, ***p < 0.001 vs. vehicle (one-way ANOVA, followed by a *post hoc* Tukey test).

Next, we examined the effects of ar-turmerone analogs on CMA-related protein expression. A2 and A4 significantly increased the levels of LAMP2A and Hsc70 at 24 h after treatment, whereas A5 and A7 did not alter these levels (Figures 6A,B). RT-qPCR revealed that treatment with A2 and A4 for 24 h significantly increased LAMP2A mRNA levels (Figure 6C). In contrast, A2 did not affect HSPA8 (Hsc70) mRNA, whereas A4 decreased it (Figure 6C), indicating that A2 and A4 upregulated LAMP2A in a transcriptional manner and Hsc70 in a post-transcriptional manner. A2 and A4 increased the mRNA levels of various lysosomal proteins, including LAMP2B, another LAMP2 variant, and LAMP1. The mRNA expression of transcription factor EB (TFEB) related to lysosome biogenesis was also induced by A2 and A4 (Figure 6C).

In addition, we investigated the protein levels of MEF2D, a CMA substrate, 24 h after chemical treatment. A2 and A4 tended to increase the expression of MEF2D, whereas MPA tended to decrease it (Figures 6A,B). However, these chemicals significantly increased MEF2D mRNA levels (Figure 6C), suggesting that the transcriptional upregulation of MEF2D masks its enhanced degradation via CMA activation by A2 and A4. To further confirm the effect of A4 on another CMA substrate, we examined the effect of A4 on the protein level of α-synuclein (Cuervo et al., 2004) in SH-SY5Y cells. A4 slightly, but significantly, reduced the protein level of α-synuclein (Supplementary Figure S3), indicating that A4 enhances the CMA-mediated degradation of α-synuclein.

### 3.5 Effects of ar-turmerone analogs on rotenone-induced cell death

**FIGURE 7:**
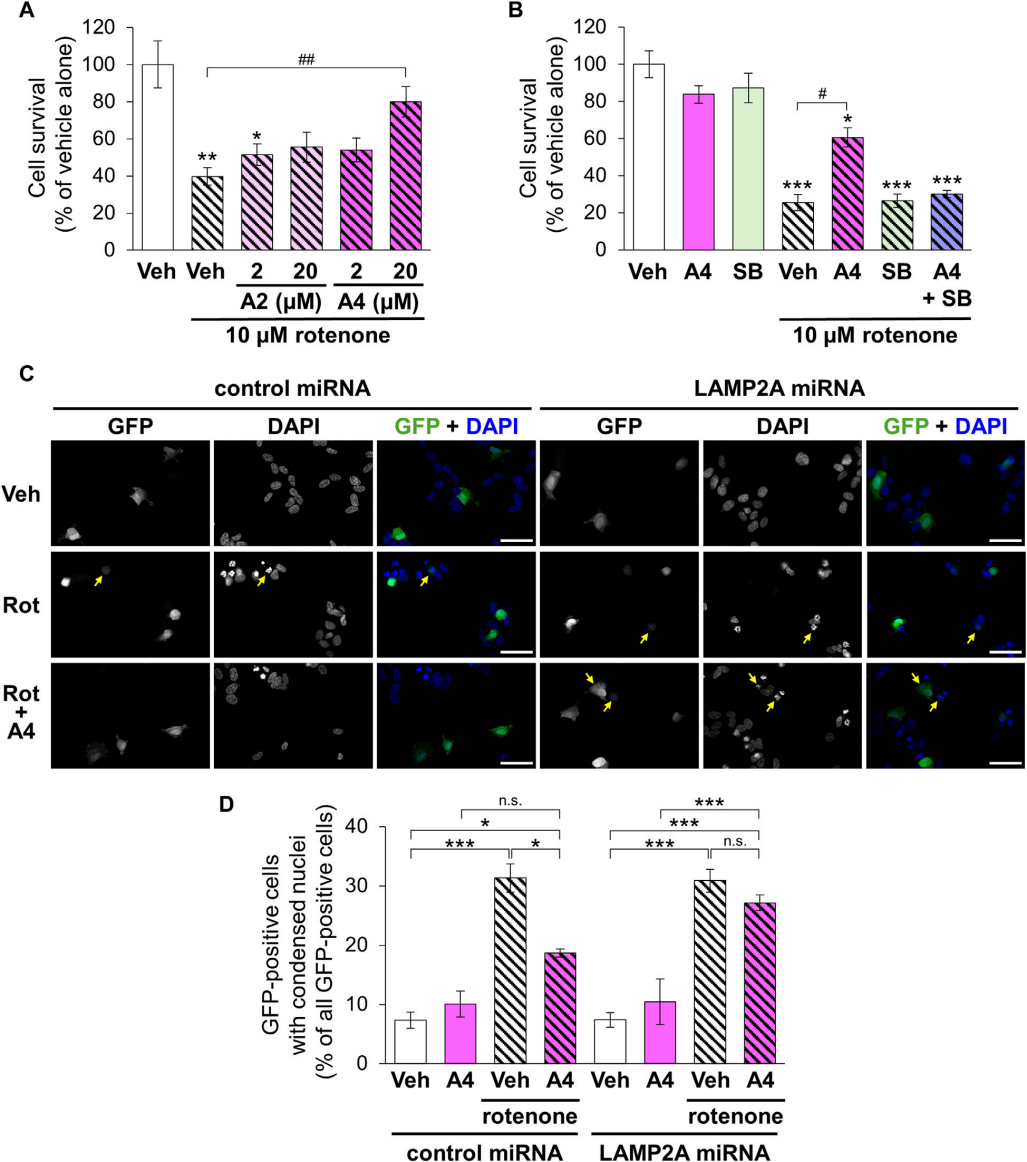
Effects of ar-turmerone analogs on the cytotoxicity of rotenone in SH-SY5Y cells stably expressing GAPDH-HT. **(A)** Survival of cells treated with vehicle and rotenone (10 μM) and co-treated with rotenone and A2 (2, 20 μM) or A4 (2, 20 μM). **(B)** Survival of cells treated with vehicle, A4 (20 μM), and/or SB203580 (SB, 10 μM) in the absence or presence of rotenone (10 μM). Data are represented as the percentage of cell survival in cells treated with vehicle alone and as the mean ± SEM of twelve **(A)** and fourteen **(B)** different samples. *p < 0.05, **p < 0.01, ***p < 0.001 vs. vehicle alone, #*p* < 0.05, ##*p* < 0.01 (Kruskal–Wallis test, followed by a *post hoc* Dunn’s test). **(C)** Representative fluorescence image of GFP and DAPI and merged images of GFP (green) and DAPI (blue) in cells transfected with plasmids to express GFP and control or LAMP2A miRNA. Cells were treated with vehicle (Veh, 0.2% DMSO), 10 μM rotenone (Rot), or rotenone and 20 μM A4 (Rot + A4) for 24 h. Yellow arrows indicate the GFP-positive cells with condensed nuclei. Scale bars = 50 μm. **(D)** Quantitative analyses of cytotoxicity indicated by the nuclear condensation. We calculated the percentage of cells with condensed nuclei in all GFP-positive cells. Data are presented as the mean ± SEM of four different wells. n.s.: not significant, *p < 0.05, ***p < 0.001 (one way ANOVA, followed by a *post hoc* Tukey test).

Rotenone is a mitochondrial toxin that inhibits the electron transport chain complex I and used for establishing animal and cellular models with PD (Betarbet et al., 2000). We evaluated the cytoprotective effect of the CMA-activating analogs A2 and A4 on rotenone-induced death of SH-SY5Y cells. Although a single treatment with A2 (20 μM) significantly enhanced the proliferation of SH-SY5Y cells, A2 (2 μM) and A4 (2 and 20 μM) did not affect their proliferation (Supplementary Figure S4). Treatment with rotenone (10 μM) significantly decreased the number of surviving cells. Although A2 did not significantly affect the cytotoxicity of rotenone, A4 (20 μM) significantly protected cells from rotenone (Figure 7A). The protective effect of A4 was diminished by the simultaneous treatment with SB205835 (Figure 7B). These results indicate that p38-mediated activation of CMA is involved in the cytoprotective effects of A4.

To validate the involvement of CMA in A4-meditated cytoprotection, we evaluated rotenone-triggered apoptosis in cells expressing negative control and LAMP2A miRNA through the observation of nuclear condensation, a hallmark of the apoptotic cell death (Van Cruchten and Van den Broeck, 2002). Nuclear staining using DAPI revealed that rotenone significantly increased cells with condensed nuclei in GFP-positive cells expressing control and LAMP2A miRNA (Figures 7C,D). Although A4 significantly reduced this increase in cells expressing control miRNA, it failed to prevent rotenone-induced apoptosis in cells expressing LAMP2A miRNA (Figures 7C,D). These findings strongly suggest that A4-induced CMA activation is related to its cytoprotective effect.

## 4 Discussion

We first identified novel ar-turmerone analogs that have a greater ability to activate Nrf2 than A2, the most potent Nrf2 activator in our previous study (Hori et al., 2021). Under normal conditions, Nrf2 interacts with Kelch-like ECH-associated protein 1 (Keap1), inducing its ubiquitination and proteasomal degradation of Nrf2. Under oxidative stress, Keap1 dissociates from Nrf2, leading to elevated protein levels and the nuclear translocation of Nrf2. It functions as a transcription factor that upregulates the expression of various antioxidant proteins, including HO-1, NQO1, and glutamate-cysteine ligase, which are related to glutathione synthesis (He et al., 2020). Keap1 contains many cysteine residues that function as sensors of oxidative stress. Electrophilic chemicals react with these cysteine residues and inhibit Nrf2-Keap1 interaction and Nrf2 activation (Ulasov et al., 2022). Based on the structure-activity relationship, we speculated that electrophilic β carbon of A2 interacts with these cysteine residues in Keap1, resulting in the dissociation of Nrf2 from Keap1 (Hori et al., 2021). Therefore, we attempted to synthesize novel ar-turmerone analogs with higher electrophilicity of β carbon through the addition of carbon-donating groups to the aromatic ring of A2 (Figure 1). All the novel chemicals had a greater ability to increase the expression of Nrf2 and its downstream targets (Figure 2). Compound A7 exhibited the most potent effect. We successfully identified novel chemicals that activate Nrf2 more potently than A2.

Nrf2 modulates CMA activity by regulating LAMP2A expression (Pajares et al., 2018). We recently demonstrated that D-cysteine activates CMA in Purkinje cells of primary cerebellar cultures through the generation of hydrogen sulfide and the activation of Nrf2 (Ueda et al., 2022). Hydrogen sulfide has been reported to trigger S-sulfhydration of cysteine residues in Keap1 and prevent its binding to Nrf2, leading to Nrf2 activation (Xie et al., 2016). Therefore, we hypothesized that chemicals that activate Nrf2 may enhance CMA activity. Indeed, we identified an ar-turmerone analog, A4, that significantly activated CMA in SH-SY5Y cells (Figure 3). However, the potency of the analogs to activate Nrf2 did not correlate with their ability to activate CMA (Figures 2, 3). In addition, the A4-mediated activation of CMA was not affected by the inhibition of Nrf2 (Figures 4A,B), indicating that Nrf2 activation is not involved in the activation of CMA induced by A4. This is inconsistent with our previous work showing that D-cysteine-induced CMA activation is prevented by the inhibition of Nrf2 (Ueda et al., 2022). Pajares et al. demonstrated that Nrf2 binds to the antioxidant response elements in LAMP2 gene and elevates mRNA expression of LAMP2A (Pajares et al., 2018). However, A7, the most potent Nrf2 activator used in this study, did not increase LAMP2A expression (Figure 6). Therefore, A7 is likely to activate other pathways that counteract Nrf2-mediated enhancement of LAMP2A expression.

Due to the experimental limitations, we could not confirm miRNA-mediated knockdown of LAMP2A and TSG101 by immunoblotting in SH-SY5Y cells. Therefore, we confirmed knockdown of these proteins in AD293 cells, derived from human embryonic kidney. We investigated whether A4 can active CMA in AD293 cells, similar to our previous studies (Sato et al., 2016; Ueda et al., 2022). However, A4 failed to increase the puncta of GAPDH-HT in AD293 cells (data not shown). Because our previous study indicated that ar-turmerone analogs directly protect dopaminergic neurons (Hori et al., 2021), we speculate that A4 activates CMA in a neuron-specific manner.

We found that persistent, but not transient, p38 activation was related to A4-induced CMA activation (Figures 4C,D). Previous studies have demonstrated that ar-turmerone reduces the phosphorylation of p38 that is enhanced by the inflammatory activation of microglial BV-2 cells triggered by the treatment with amyloid β and lipopolysaccharide (Park et al., 2012a; 2012c). In contrast, ar-turmerone does not affect p38 phosphorylation triggered by 12-O-tetradecanoylphorbol-13-acetate (TPA) in breast cancer cells (Park et al., 2012b). However, ar-turmerone enhanced the proliferation and differentiation of neural stem cells (Hucklenbroich et al., 2014). Because p38 participates in the differentiation of neural stem cells (Fernando et al., 2005; Kase et al., 2019), it is possible that p38 activation by ar-turmerone is related to the enhancement of neural differentiation. Taken together, these findings suggest that ar-turmerone does not directly interact with p38 but affects its upstream targets in a cell type-dependent manner.

The enhancement of p38 phosphorylation by ar-turmerone analogs was parallel to the increase in Nrf2 6 h after stimulation (Figures 6A,B), suggesting that electrophilicity determines the potential of ar-turmerone analogs to activate p38. Thioredoxin (Trx) and thioredoxin reductase (TrxR) are involved in the regulation of the cellular redox status. Trx and TrxR contain several cysteine and selenocysteine residues important for redox regulation. The cysteine residue of TrxR is modified by curcumin (Cai et al., 2012), which interacts with Keap1 via its cysteine residues to stabilize Nrf2 (Shin et al., 2020). Trx modulates the activities of apoptosis signal-regulating kinase 1 (ASK1) and MAP three kinase 1 (MTK1), which are upstream kinases of the p38 pathway (Liu and Min, 2002). Therefore, dysregulation of Trx and TrxR through the modification of cysteine residues may be a molecular mechanism by which ar-turmerone analogs activate p38.

We revealed that persistent activation for 24 h of p38 is crucial for CMA activation. Under ER stress, p38 directly phosphorylates LAMP2A and stabilizes its oligomers in the lysosomal membrane, leading to CMA activation (Li et al., 2017). In the present study, immunostaining revealed that phosphorylated p38 was mainly present in the nuclei of SH-SY5Y cells (Figures 5E,F). A4-induced activation of p38 enhances its nuclear localization. We did not detect lysosomal accumulation of phosphorylated p38 following A4 treatment (data not shown). Nuclear translocation of p38 is frequently observed under various stress conditions and regulates various transcription factors via phosphorylation (Maik-Rachline et al., 2020). Therefore, A4-induced elevation of TEFB and MEF2D mRNA levels may be due to nuclear p38. We hypothesized that persistent activation of p38 is necessary for the transcriptional alteration of several mRNAs. In the current study, we identified a novel mechanism by which p38 activates CMA in a manner different from that described in a previous report (Li et al., 2017).

We found that A2 and A4 increased the levels of LAMP2A and Hsc70, which are CMA-related proteins (Figures 5A,B). LAMP2A expression increased at the transcriptional level (Figure 5C). Together with LAMP2B and LAMP1, LAMP2A mRNA expression is upregulated by TFEB, which is also transcriptionally upregulated. In addition to transcriptional regulation, TFEB may also be activated by p38-mediated phosphorylation (Martina et al., 2023). However, according to the analysis of the ratio of LAMP2A and LAMP2B to LAMP1, LAMP2A mRNA was highly upregulated compared to LAMP1, whereas LAMP2B was not (Supplementary Figure S5). Because LAMP2A and 2B are splicing variants, p38 may affect the splicing of LAMP2. p38 regulates alternative splicing via the phosphorylation of SNW domain-containing protein 1, a component of the spliceosome (Carbonell et al., 2019). Therefore, it is possible that p38 is involved in the regulation of the alternative splicing of LAMP2. Although the detail molecular mechanism of alternative splicing of LAMP2 remains unknown, pleckstrin homology, and RUN domain-containing M2 (PLEKHM2) participates to the splicing (Muhammad et al., 2015). Whether p38 interacts with and affects the function of PLEKHM2 has not yet been elucidated. Further studies are necessary to reveal the relationship between p38 and the alternative splicing of LAMP2.

In contrast to LAMP2A, A2 and A4 did not induce the upregulation of HSPA8 (Hsc70) mRNA (Figure 6C), indicating that the elevation of Hsc70 protein by A2 and A4 was due to post-transcriptional modulation. A previous report revealed that metformin activates CMA via the phosphorylation of Ser72 of Hsc70 by IκB kinase (Xu et al., 2021); however, this phosphorylation does not affect the expression level of Hsc70. Although the relationship between p38 and the phosphorylation of Hsc70 remains unknown, alterations in Hsc70 phosphorylation might affect its protein levels in the presence of A2 and A4. Ubiquitylome and acetylome analyses have revealed that ubiquitination and acetylation of Hsc70 are increased in A549 cells treated with a histone deacetylase (HDAC) inhibitor (Wu et al., 2015). In addition, the overexpression of sirtuin 3, a protein deacetylase, activates CMA by elevating Hsc70 levels in hepatocytes treated with fatty acids (Zhang et al., 2019). Furthermore, HDAC6 inhibitors increase the levels of Hsc70 in the mouse brain (Sreenivasmurthy et al., 2022). These findings suggest that changes in acetylation may affect the stability of Hsc70. Further studies are required to determine whether A4 affects HDAC activity by regulating Hsc70 levels.

A cell survival assay revealed that A4 had a higher cytoprotective activity against rotenone toxicity than A2 (Figure 7A). This protective potency correlated with the potency of CMA activation (Figure 3). The role of CMA was confirmed by the loss of cytoprotection in the presence of a p38 inhibitor (Figure 7B). Because reactive oxygen species are crucial for the toxicity of rotenone (Alkholifi and Albers, 2015), Nrf2-mediated elevation of antioxidant proteins might mediate the cytoprotective effect of A4. However, ML385 did not affect the protective effects of A4 (Supplementary Figure S6). Therefore, p38-mediated CMA activation mainly contributes to the cytoprotective effects of A4. Sala et al. (2016) demonstrated that rotenone reduces Hsc70, leading to the impairment of CMA and the accumulation of α-synuclein. Although we did not examine the levels of Hsc70 and α-synuclein, we confirmed that A4 significantly increased the protein level of Hsc70 (Figure 6). In addition, the protective effect of A4 against rotenone-induced toxicity was diminished in cells expressing LAMP2A miRNA (Figures 7C,D). These findings strongly suggest that the protective effect of A4 is caused by the CMA activation through the increases in Hsc70 and LAMP2A. Several studies have indicated that CMA activation by chemicals or LAMP2A overexpression protects cells from toxic stimuli (Anguiano et al., 2013; Ghosh et al., 2022). Additionally, CMA is activated under oxidative stress and contributes to the degradation of oxidized proteins (Kiffin et al., 2004). Collectively, these findings suggest that CMA is crucial for protecting cells from toxicity and oxidative stress.

We focused on A4 as a CMA activator in this study. Regarding Nrf2, A7 is a more potent activator of Nrf2 than A4 and other ar-turmerone analogs. Since Nrf2 induces the expression of several anti-oxidative proteins (He et al., 2020), it is considered as a therapeutic target for various chronic diseases, including neurodegenerative diseases (Cuadrado et al., 2019; Brandes and Gray, 2020). Therefore, A7 is a valuable candidate therapeutic agent for various diseases.

In the present study, we identified a novel ar-turmerone analog (A4) that enhances CMA activity via persistent activation of p38, and elucidated a novel mechanism by which nuclear-localized p38 regulates CMA. As described in the introduction, a decline in CMA activity is frequently observed in models of various neurodegenerative diseases (Campbell et al., 2018; Bourdenx et al., 2021; Kanno et al., 2022; Idera et al., 2023). Therefore, CMA may be a novel therapeutic or preventive target for various neurodegenerative diseases. Indeed, overexpression of several CMA-activating chemicals and LAMP2A ameliorated the phenotypes of mouse models of several neurodegenerative diseases (Xilouri et al., 2013; Bourdenx et al., 2021; Ohta et al., 2021; Xu et al., 2021; Sreenivasmurthy et al., 2022). The novel ar-turmerone analog, A4, could be a therapeutic or preventive candidate for neurodegenerative diseases through the activation of CMA.

## Data Availability

The raw data supporting the conclusions of this article will be made available by the authors, without undue reservation.
