# Supplementary material for: Identification of a novel aromatic-turmerone analog that activates chaperone-mediated autophagy through the persistent activation of p38
Source: Front Cell Dev Biol. 2024 Aug 8;12:1418296. doi: 10.3389/fcell.2024.1418296 (PMC11342337; doi:10.3389/fcell.2024.1418296)
Supplement: Supplementary file 1 [file DataSheet1.PDF]

Supplementary Material

1 Supplementary methods

AD293 cells were purchased from Riken Cell Bank (Tsukuba, Japan) and cultured in Dulbecco's modified Eagle medium/F-12 mixture (1:1) medium, supplemented with 10 % fetal bovine serum (FBS), 100 units/ml penicillin, and 100 μg/ml streptomycin in a humidified atmosphere containing 5 % CO_2 at 37 °C. Cells were transfected with plasmids to express GFP and miRNA (control, LAMP2A, and TSG101) using K4 transfection system. Transfected cells were spread onto the 8 well coverglass chamber and 12 well plates for the observation using a fluorescence microscopy and immunoblotting, respectively. Three days after the transfection, cells on coverglass chamber were fixed with 4 % PFA, followed by the DAPI staining. Fluorescence images of GFP and DAPI were obtained using THUNDER imaging system. Cells on 12 well plates were harvested in RIPA buffer, followed by the immunoblot analyses using anti-LAMP2A, TSG101, and β-actin antibodies.

2 Supplementary Figures

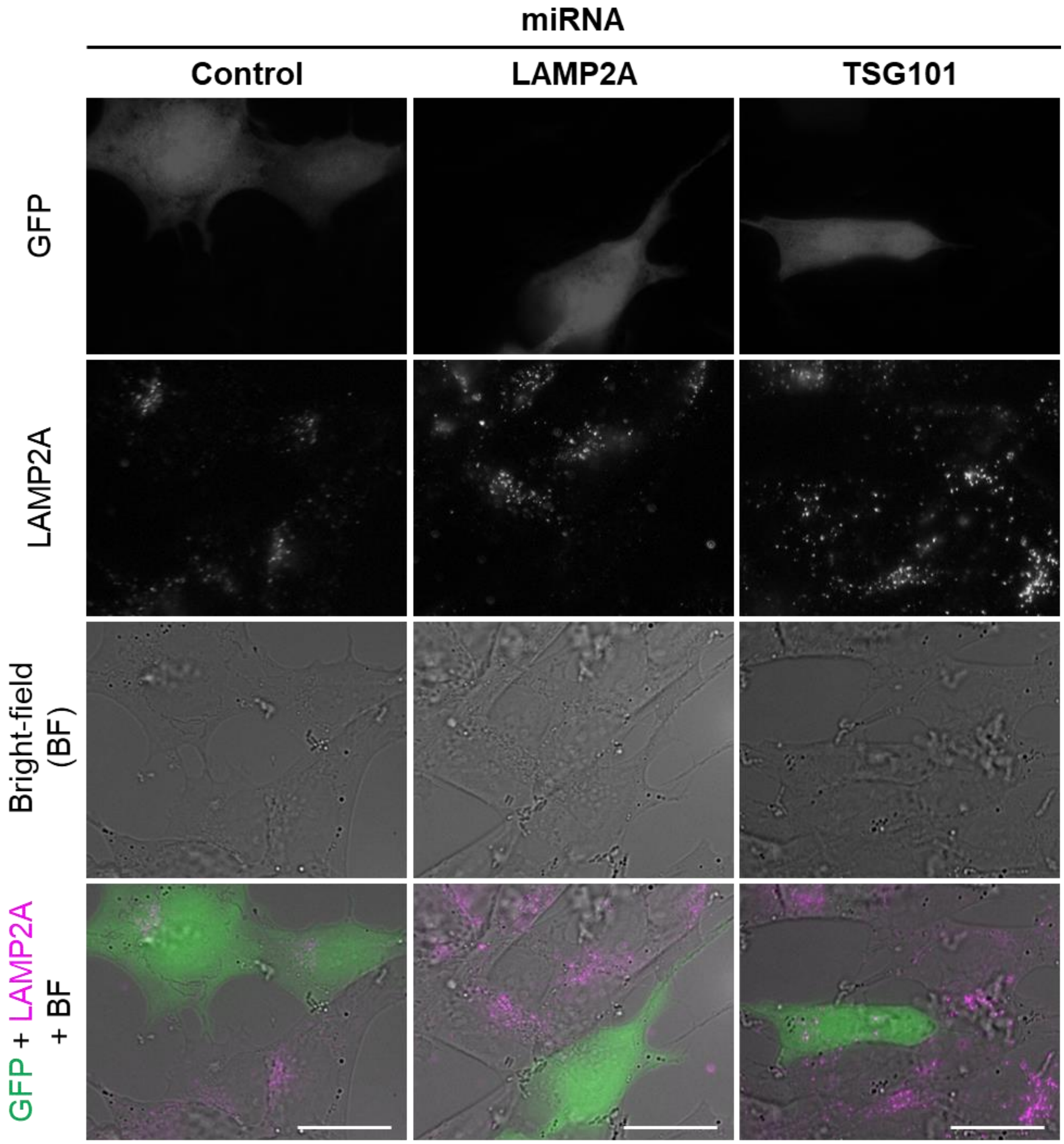

Supplementary figure 1. Confirmation of miRNA-mediated knockdown of LAMP2A in SH-SY5Y cells.

Representative images of GFP fluorescence, LAMP2A immunofluorescence, bright-field (BF), and merged images of GFP (green), LAMP2A immunofluorescence (magenta), and BF (gray) in SH-SY5Y cells expressing GFP and miRNA (control, LAMP2A, and TSG101). Scale bars = 20 μm.

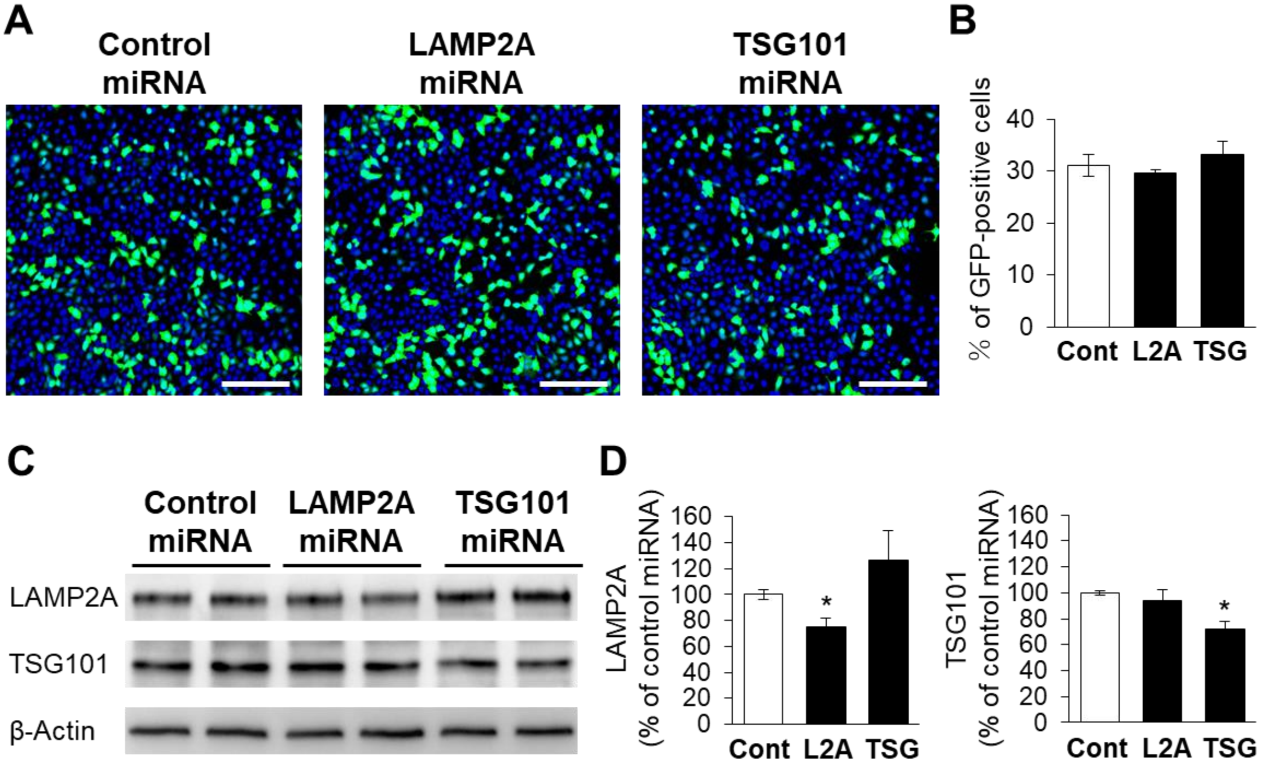

Supplementary figure 2. Confirmation of miRNA-mediated knockdown in AD293 cells.

(A) Representative merged images of GFP and DAPI in AD293 cells expressing GFP and miRNA (control, LAMP2A, and TSG101). Scale bars = 100 µm. (B) Quantitative analyses of the ratio of GFP-positive cells in DAPI-stained cells. Data are presented as the mean ± SEM of four different samples. (C) Representative immunoblot images of LAMP2A, TSG101, and β-actin in cells transfected with control, LAMP2A, or TSG101 miRNAs. (D) Quantitative analyses of the immunoreactive bands of LAMP2A and TSG101. The band intensities were normalized with the bands of β-actin as an internal control. Data are presented as the mean ± SEM of four different samples. * $p < 0.05$ vs cells transfected with control miRNA (one-way ANOVA, followed by a post hoc Tukey test).

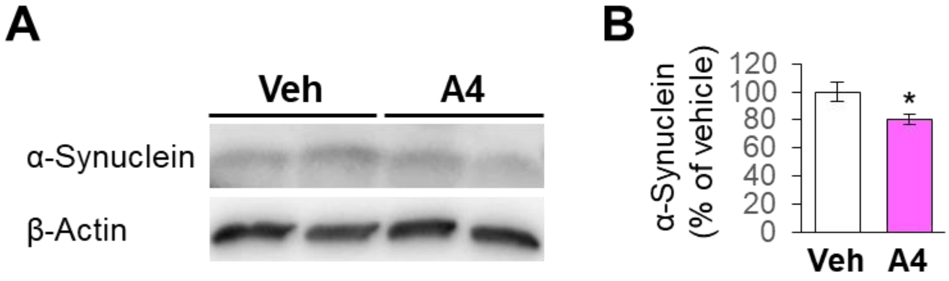

Supplementary figure 3. Confirmation of miRNA-mediated knockdown in AD293 cells.

(A) Representative immunoblot images of α-synuclein and β-actin in cells treated with vehicle or A4 (20 μM) for 24 h. (B) Quantitative analyses of the immunoreactive bands of α-synuclein. The band intensities were normalized with the bands of β-actin as an internal control. Data are presented as the mean ± SEM of four different samples. * $p < 0.05$ vs cells transfected with control miRNA (unpaired t-test).

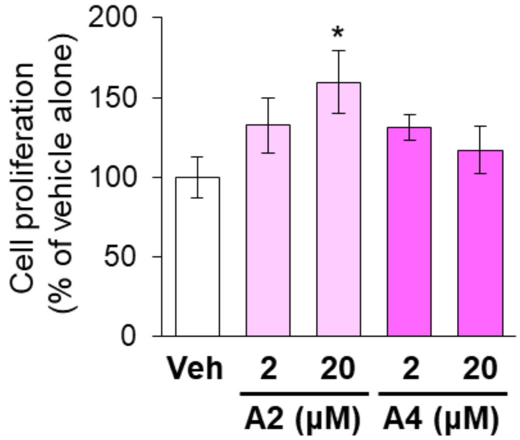

Supplementary figure 4. Effects of single treatments of ar-turmerone analogs on the proliferation of SH-SY5Y cells

The proliferation rate of SH-SY5Y cells treated with vehicle, A2, and A4 (2, 20 μM), evaluated using the cell counting kit-8. Data are represented as the percentage of cell survival in cells treated with vehicle alone and as the mean ± SEM of twelve different samples. * $p < 0.05$, vs. vehicle (Kruskal-Wallis test, followed by a post hoc Dunn's test).

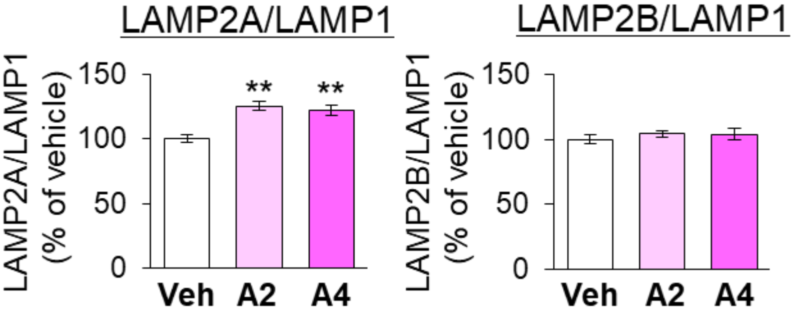

Supplementary figure 5. Ratios of LAMP2A and LAMP2B mRNAs per LAMP1 mRNA.

Ratios of LAMP2A and LAMP2B mRNAs per LAMP1 mRNA were calculated using the Ct values of the same samples obtained from RT-qPCR analyses shown in Fig. 6C. Data are presented as the mean ± SEM of four different samples. ** $p < 0.01$ vs cells transfected with control miRNA (one-way ANOVA, followed by a post hoc Tukey test).

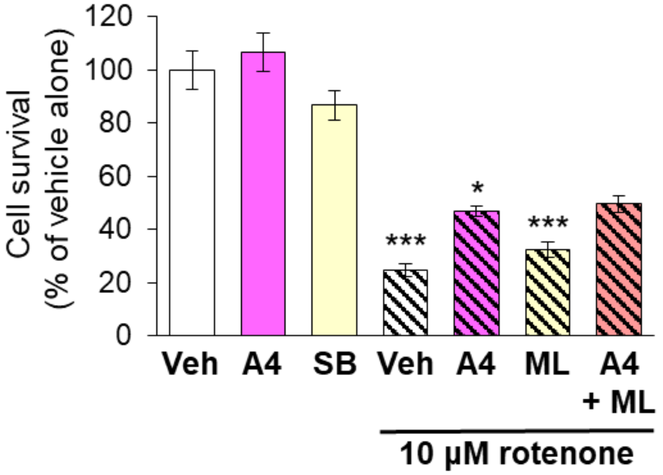

Supplementary figure 6. Effects of an Nrf2 inhibitor on the cytoprotection of A4 in SH-SY5Y cells stably expressing GAPDH-HT.

Survival of cells treated with vehicle (Veh), A4 (20 μM), and/or ML385 (ML, 1 μM) in the absence or presence of rotenone (10 μM). Data are represented as the percentage of cell survival in cells treated with vehicle alone and as the mean ± SEM of eleven different samples. * $p < 0.05$, *** $p < 0.001$ vs. vehicle alone (Kruskal-Wallis test, followed by a post hoc Dunn's test).
